# Significant increase of serum extracellular vesicle-packaged growth differentiation factor 15 in type 2 diabetes mellitus: a cross-sectional study

**DOI:** 10.1186/s40001-023-01009-6

**Published:** 2023-01-19

**Authors:** Wen Zhao, Xinwei Li, Xinxin Li, Lu Peng, Yu Li, Yunhui Du, Jianxun He, Yanwen Qin, Huina Zhang

**Affiliations:** 1 Beijing Anzhen Hospital, Beijing Institute of Heart Lung and Blood Vessel Disease, Capital Medical University, No. 2 Anzhen Road, Beijing, 100029 China; 2 Beijing Anzhen Hospital Laboratory Department, Beijing Anzhen Hospital, Capital Medical University, Beijing, 100029 China

**Keywords:** Type 2 diabetes mellitus, Extracellular vesicles, Growth differentiation factor 15, Serum, Biomarker

## Abstract

**Background:**

Growth differentiation factor 15 (GDF15) is a stress-inducible factor involved in the inflammatory progression of many complications, including type 2 diabetes mellitus (T2DM). Growing evidence suggests that molecules in extracellular vesicles (EVs) are associated with diabetes or diabetes-related complications. However, the correlation between serum extracellular vesicle-derived growth differentiation factor15 (EV-GDF15) and T2DM is unknown. The aim of this cross-sectional study is to investigate whether serum EV-GDF15 is associated with T2DM incidence.

**Methods:**

116 individuals, including 78 T2DM and 38 non-T2DM, were recruited as participants. The concentrations of serum EV-GDF15 and serum GDF15 were determined by Luminex assay. Serum EVs were obtained by ultracentrifugation. Multivariate stepwise regression analysis was used to determine the association between serum GDF15 levels and fasting plasma glucose (FPG) as well as glycated hemoglobin (HbA1c). The association of serum EV-GDF15 levels with T2DM was determined by multivariate logistic regression analysis.

**Results:**

Our data showed that the levels of serum EV-GDF15 and serum GDF15 were significantly increased in T2DM patients compared with non-T2DM subjects (EV-GDF15 levels, 13.68 (6.61–23.44) pg/mL vs. 5.56 (3.44–12.09) pg/mL, *P* < 0.001; and serum GDF15 levels, 1025.49 (677.87–1626.36) pg/mL vs. 675.46 (469.53–919.98) pg/mL, *P* < 0.001). There was a linear correlation between EV-GDF15 levels and fasting plasma glucose (FPG) and Hemoglobin A1C (HbA1c) levels (normalized *β* = 0.357, *P* < 0.001; normalized *β* = 0.409, *P* < 0.001, respectively). Elevated levels of EV-GDF15 were accompanied by an increase in the proportion of patients with T2DM (from 47.5 to 78.9%) and a progressive independent association with the incidence of T2DM (from OR = 3.06, 95% CI 1.02–9.19, *P* = 0.047 to OR = 3.75, 95% CI 1.14–12.26, *P* = 0.029). Notably, high levels of serum GDF15 plus high levels of serum EV-GDF15 were significantly associated with T2DM more than either alone.

**Conclusion:**

This study elucidated that increased levels of GDF15 in serum EVs were independently associated with T2DM.

**Supplementary Information:**

The online version contains supplementary material available at 10.1186/s40001-023-01009-6.

## Background

Type 2 diabetes mellitus (T2DM) is the main reason leading to cardiovascular diseases that cause considerable morbidity and mortality worldwide [1]. High fasting plasma glucose (FPG) and elevated levels of Hemoglobin A1C (HbA1c) are the most commonly used indicators for the clinical diagnosis of diabetes. However, these two indicators are susceptible to external factors and do not accurately reflect the course of diabetes and its associated complications. Therefore, finding biomarkers that can more sensitively respond to the characteristics of the occurrence and development of diabetes has become a research hotspot in recent years. Studies have shown that acute phase proteins (C-reactive protein, fibrinogen activator inhibitor-1) and coagulation factors (fibrinogen, d-dimer) can be used as predictors of prediabetes [2]. In addition, extensive research have also addressed the relevance of other inflammatory factors and metabolic molecules, such as omentin [3], neuregulin [4], and the ratio of serum uric acid to HDL-cholesterol [5] to T2DM. However, these factors lack disease specificity in clinical application. Therefore, there is still a long way to go in the search of diabetes-related biomarkers.

Extracellular vesicles (EVs) nowadays have attracted wide attention in the field of humoral biopsy since its levels and molecular cargoes are closely relevant to some specific diseases. For example, EVs have been well studied as a potential clinical biomarker in inflammation-related diseases [6, 7] as well as in cancers [8]. In the field of diabetes, researchers have paid more attention to the relationship between diabetes or diabetes-related syndromes and EVs levels with different origins. For instance, a longitudinal cohort study proved that circulating EVs levels, especially levels of erythrocyte-derived EVs, were elevated in patients who progressed to diabetes and were positively correlated with homeostasis model assessment insulin resistance (HOMA-IR) [9]. The levels of urine-derived extracellular vesicles (uEVs) were higher in diabetic nephropathy patients compared with controls and were inversely correlated with the assessed glomerular filtration rate (eGFR) [10]. Our group recently reported the significant association of elevated serum extracellular vesicle-derived myeloperoxidase (EV-MPO) and serum extracellular vesicle-derived Arginase 1 (EV-ARG1) with T2DM incidence [11, 12]. The exploration of the clinical significance of EVs contents in relation to diabetes and its complications is still in its infancy, although a number of basic research has addressed the involvement of EVs contents, including miRNAs (miR-326 [13], miR-877-3p, and miR-150-5p [14]) and proteins (IRS1 [15] and ARG1 [16]) in the development of diabetes-related pathophysiological disorders.

Growth differentiation factor 15 (GDF15), a member of TGF-β/BMP superfamily, is a stress response cytokine released into the blood after tissue injury, hypoxia, and proinflammatory response. Almost all cells have the ability to secrete GDF15, for example, it can be released from macrophages [17], vascular smooth muscle cells [18], cardiomyocytes, adipocytes [19], and endothelial cells [20]. Under normal conditions, the circulating levels of GDF15 were 0.1–1.2 ng/mL in serum [21], while its levels are significantly elevated in obesity, non-alcoholic fatty liver disease, cardiovascular disease, and cancer cachexia [22]. Circulating GDF15 levels were also elevated in patients with T2DM and positively correlated with FPG levels [23]. Studies have shown that GDF15 can increase insulin sensitivity in mice fed a high-fat diet, suggesting its potential in the treatment of T2DM [24]. However, changes in GDF15 levels in EV and their clinical relevance to T2DM are unclear and need to be explored in depth.

In the present study, the levels of GDF15 in both serum and serum EV in T2DM patients and non-T2DM subjects were detected by Luminex assay. And the association of serum GDF15 and serum EV-GDF15 with T2DM was analyzed.

## Methods

### Study design and participants

The cross-sectional study was conducted at Beijing Anzhen Hospital affiliated to Capital Medical University, from August 2018 to March 2019. All participants had written informed consent prior to enrollment. The protocol was approved by the Ethics Committee of Beijing Anzhen Hospital (No. 2017005) and complied with the Declaration of Helsinki.

In this study, 251 patients were recruited in the emergency department of Beijing Anzhen Hospital as previous reported [11]. The diagnosis of patients with T2DM is based on the American Diabetes Association criteria of FPG (fasting plasma glucose) ≥ 126 mg/dL (7.0 mmol/L), 2-h PG (2-h plasma glucose) ≥ 200 mg/dL (11.1 mmol/L) during OGTT (oral glucose tolerance test), HbA1c (glycated hemoglobin) ≥ 6.5%, or random plasma glucose ≥ 200 mg/dL (11.1 mmol/L) in patients with typical symptoms of hyperglycemia or hyperglycemic crisis [25]. Subjects were excluded if they had type 1 diabetes, various cancer-related diseases, kidney or liver diseases, acute or chronic inflammatory diseases, presence of hepatitis C, alcohol or drug abuse, thyroid dysfunction, acute or chronic infectious diseases, or any hematologic diseases; female subjects who were taking hormone replacement therapy were also excluded. Subjects under the age of 18 and pregnant women were also excluded. We also excluded patients with stroke, angina, and heart failure, which might affect GDF15 levels. A final 116 participants (78 T2DM patients and 38 non-T2DM subjects) were included. The flowchart of patient selection is demonstrated in Additional file 1: Fig. S1.

### Demographic data collection

All subjects were screened for medical history (i.e., age, smoking, alcohol consumption, and treatment). Height and weight were measured wearing light clothing and no shoes, and blood pressure was measured while sitting by the same observer. Body mass index (BMI) was calculated as weight (kg) divided by the square of height (m). Plasma glucose was measured by the glucose oxidase method. Non-smokers were defined as individuals who had never smoked or who had not smoked for at least 1 year prior to enrollment. The remaining subjects were classified as smokers. Alcohol consumption was defined as consuming alcohol ≥ 3 times per week for ≥ 1 year.

### Blood sample preparation

Blood samples were taken from all patients after overnight fasting. Blood serum was obtained by centrifugation at 3000 rpm for 10 min, then equally divided, and stored in – 80 ℃ for subsequent analysis. Serum triglycerides (TG), total cholesterol (TC), low-density lipoprotein cholesterol (LDL-C), high-density lipoprotein cholesterol (HDL-C), high-sensitivity C-reactive protein (hs-CRP), fasting plasma glucose (FPG), glycated hemoglobin (HbA1c), and homocysteine (Hcy) were measured by biochemical analyzer (Hitachi 7600, Tokyo, Japan) in the clinical laboratory of Beijing Anzhen Hospital.

### Serum EVs purification and identification

Serum EVs were isolated and purified from serum by differential centrifugation. 700 μL serum diluted with an equal volume of phosphate buffer salt solution (PBS) was centrifuged at 2000 ×*g* for 10 min and then 12,000 ×*g* for 45 min to eliminate dead cells and cell debris. The supernatant was transferred to ultracentrifuge tubes and ultracentrifuged at 110,000 ×*g* for 70 min (Beckman Coulter, CA, USA). All pellets were resuspended in PBS and ultracentrifuged again at 110,000 ×*g* for 70 min. All centrifugations were performed at 4 ℃. The ultrastructure of serum EVs was observed by transmission electron microscopy after negative staining (TEM, Hitachi H-7650, Tokyo, Japan). The diameter and concentrations of EVs were measured by nanoparticle tracking analysis (NTA) (Malvern, UK). EV pellets were resuspended in 200 μL PBS with a protease inhibitor cocktail (Thermo Fisher Scientific, cat#: 87785) and subjected to five times freeze–thaw cycles (− 17 to 37 ℃) to rupture the membrane and release the EV proteins for Luminex assays.

### Western blotting

The expression of EV marker proteins was detected by Western blotting. Protein samples prepared from serum, EV, or EV-free serum were separated by sodium dodecyl sulfate–polyacrylamide gels and transferred onto polyvinylidene fluoride membranes. The membranes were blocked with 5% fat-free dried milk in TBST at room temperature for 1 h and probed with the primary antibodies [anti-CD63 (EXOAB-CD63A-1, 1:1000, System Biosciences, Palo Alto, CA, USA), anti-CD81 (EXOAB-CD81A-1, 1:1000, System Biosciences, Palo Alto, CA, USA), and anti-TSG101 (EXOAB-TSG101-1, 1:1000, System Biosciences, Palo Alto, CA, USA)] overnight at 4 °C. After 2 h incubation with specific horseradish peroxidase-conjugated secondary antibodies, enhanced chemiluminescence (Thermo Fisher Scientific, MA, USA) was used to detect the protein expression levels.

### Luminex assays

EV-GDF15 and serum GDF15 levels were measured by Luminex assays according to the manufacturer’s instructions. EV proteins were dissolved in 200 μL PBS as Luminex test samples. A mixture of Halt protease inhibitor cocktail (Thermo Fisher Scientific, cat#: 87786) was then added to a PBS medium containing EV proteins to protect the proteins from degradation. Then, according to the manufacturer's instructions, serum GDF15 levels and EV-GDF15 were measured using Luminex-based Multiplex panels (R&D Systems, Minneapolis, MN, USA).

### Statistical analysis

Statistical analysis of the data was performed using the Statistical Package for the Social Sciences, version 25.0 (SPSS, Inc., Chicago, IL, USA). The continuous variables of normal distribution are expressed as mean ± standard deviation (SD) and tested with one-way ANOVA by tertiles of EV-GDF15. Kolmogorov–Smirnov test (for data with a sample size greater than 50) and Shapiro–Wilk test (for data with a sample size less than 50) were used to determine whether the relevant data were normally distributed. Continuous variables of skewed distribution are expressed as median and inter-tertile ranges (25–75th percentile) and analyzed with Kruskal–Wallis test. The Chi-square test was used to analyze the difference in categorical variables. Spearman correlation test was used to determine the correlation between EV-GDF15 and other clinical variables. The association between T2DM and serum EV-GDF15 levels was explored in a multivariable logistic regression model. Subgroup analysis and interaction test were used to evaluate the interaction of the relevant baselines on the association between EV-GDF15 levels and T2DM. A likelihood ratio test was used to calculate the interaction *P*-value. *P*-value less than 0.05 was considered statistically significant.

## Results

### Characteristics of EVs

**Fig. 1:**
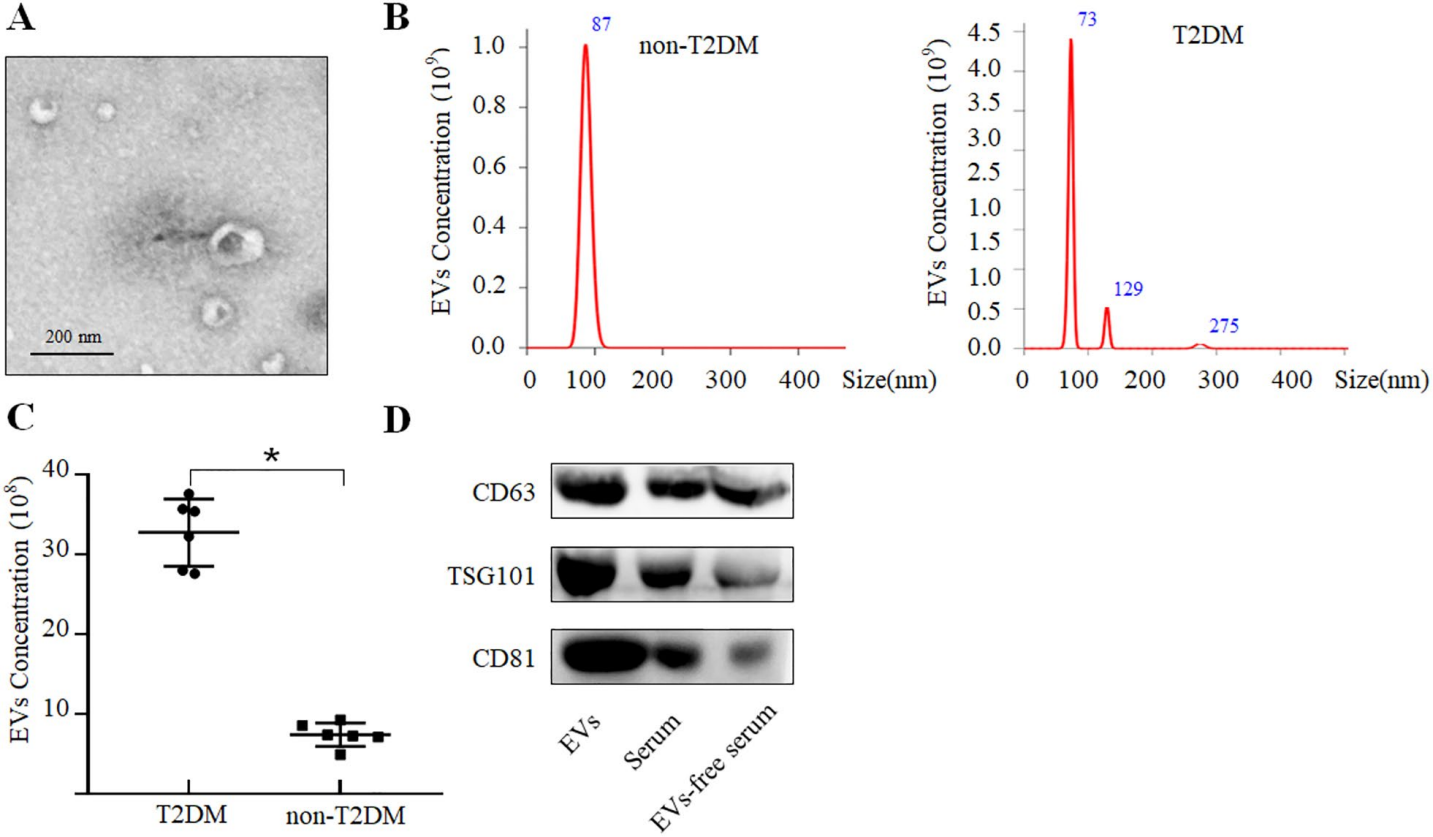
Characterization of EVs derived from T2DM and non-T2DM serum. **A** Electron microscopy image of EVs isolated from serum. Scale bar = 200 nm. **B** The concentration and size distribution of serum EVs from T2DM and non-T2DM subjects were measured by nanoparticle tracking analysis (NTA). **C** The levels of EVs from T2DM and non-T2DM patients were detected by NTA (*n* = 6). **D** Western blotting results showed the expression of CD63, CD81, and TSG101 in different protein fractions. *T2DM* type 2 diabetes mellitus, *EVs* extracellular vesicles, *NTA* nanoparticle tracking analysis. *P < 0.05 vs. non-T2DM

Firstly, we evaluated the characteristics of serum-derived EVs obtained by ultracentrifugation. EVs appeared typically spherical as observed by transmission electron microscopy (Fig. 1A). According to the nanoparticle tracking analysis (NTA) results, the diameters of the serum EVs from non-T2DM and T2DM were very similar and were concentrated in the range of 50–130 nm, with approximately 87.0 ± 39.5 nm in non-T2DM and 84.2 ± 37.1 nm in T2DM (Fig. 1B). EV concentrations in T2DM were significantly higher than those in non-T2DM group [(32.77 ± 4.21) × 10^8^/mL vs. (7.42 ± 1.48) × 10^8^/mL, *P* = 0.002) (Fig. 1C). Western blotting results showed that the expression of CD63, CD81, TSG101 (markers of EV) was enriched in EV subfractions compared to total serum as well as EV-free serum (Fig. 1D and Additional file 1: Fig. S2).

### Clinical characteristics of the participants

A total of 116 patients with a median age of 61.0 (56.0–67.8) years were recruited in the study, including 78 T2DM patients and 38 non-T2DM participants. Three subgroups were divided based on tertile levels of EV-GDF15. Baseline characteristics demonstrated that FPG and HbA1c levels as well as age, TC, and LDL-C were statistically different among the three groups (Table 1). Along with the increase of EV-GDF15 levels in the three subgroups [< 6.01 pg/mL (*n* = 40); < 17.25 pg/mL (*n* = 38); ≥ 17.25 pg/mL (*n* = 38)], the FPG levels were increased from 5.52 mmol/L in the first group [< 6.01 pg/mL (*n* = 40)] to 9.66 mmol/L in the group with the highest EV-GDF15 levels [≥ 17.25 pg/mL (*n* = 38)] (P < 0.01). Similarly, with the increase of EV-GDF15 levels, HbA1c levels increased from 5.80 to 8.30% (*P* < 0.01) (Table 1 and Additional file 1: Fig. S3).

**Table 1:** The baseline characteristics of the patients by levels of EV-GDF15

	Total (*n* = 116)	< 6.01(pg/mL) (*n* = 40)	< 17.25 (pg/mL) (*n* = 38)	≥ 17.25 (pg/mL) (*n* = 38)	*P*-value
Age (years)	61.0 (56.0–67.8)	61.0 (53.0–67.0)	64.5 (59.0–68.5)	59.0 (52.0–68.0)	0.010*
Male (*n*, %)	77 (66.4%)	27 (67.5%)	25 (65.8%)	25 (65.8%)	0.245
BMI (kg/m^2^)	26.35 (23.62–30.07)	27.00 (24.36–30.36)	25.63 (22.69–27.79)	27.54 (25.52–30.24)	0.146
FPG (mmol/L)	6.07 (5.42–8.95)	5.52 (5.19–5.92)	6.20 (5.29–7.76)	9.66 (6.67–12.96)	< 0.001**
HbA1c (%)	6.80 (5.80–7.90)	5.80 (5.40–6.50)	6.90 (6.25–7.85)	8.30 (6.78–10.28)	< 0.001**
SBP (mmHg)	130.0 (124.0–141.5)	130.0 (121.3–140.0)	130.0 (121.5–136.3)	138.0 (126.6–148.0)	0.113
DBP (mmHg)	77.0 (69.0–86.8)	77.0 (70.0–80.8)	74.0 (65.8–88.0)	80.0 (68.8–90.0)	0.612
Smoker (*n*, %)	50 (43.1%)	18 (45.0%)	16 (42.1%)	16 (42.1%)	0.148
Drinker (*n*, %)	32 (27.6%)	11 (28.9%)	10 (26.3%)	11 (28.9%)	0.819
TG (mmol/L)	1.42 (1.03–1.84)	1.52 (1.29–1.85)	1.38 (0.99–1.76)	1.31 (1.00–1.81)	0.789
TC (mmol/L)	3.73 (3.14–4.57)	4.16 (3.33–4.83)	3.71 (3.26–4.59)	3.39 (3.09–3.98)	< 0.001**
LDL-C (mmol/L)	2.30 ± 0.90	2.44 ± 0.90	2.34 ± 0.91	2.11 ± 0.88	0.001*
HDL-C (mmol/L)	1.07 ± 0.34	1.13 ± 0.39	1.07 ± 0.28	1.00 ± 0.32	0.052
Hcy (μmol/L)	11.80 (10.33–14.45)	11.45 (10.07–13.03)	11.90 (10.28–14.60)	12.85 (10.58–15.35)	0.069
hs-CRP (mg/L)	1.34 (0.58–4.98)	1.34 (0.56–3.17)	0.76 (0.46–2.31)	3.00 (0.80–7.08)	0.693
EV-GDF15 (pg/mL)	10.48 (4.82–20.86)	3.55 (2.75–5.11)	10.77 (8.39–13.82)	28.18 (20.86–52.66)	< 0.001**
Serum GDF15 (pg/mL)	878.50 (614.98–1375.07)	646.98 (449.12–988.67)	878.50 (657.40–1310.82)	1161.18 (742.12–1919.63)	< 0.001**

Results are expressed as mean ± SD, median (interquartile range), or *n* (%). Differences between groups were analyzed by one-way ANOVA test, *χ*^2^ text, or Kruskal–Wallis test

*T2DM* type 2 diabetes mellitus, *BMI* body mass index, *SBP* systolic blood pressure, *DBP* diastolic blood pressure, *FPG* fasting plasma glucose, *HbA1c* glycated hemoglobin, *TG* triglycerides, *TC* total cholesterol, *LDL-C* low-density lipoprotein cholesterol, *HDL-C* high-density lipoprotein cholesterol, *Hcy* homocysteine, *hs-CRP* high-sensitive C-reactive protein, *EV* extracellular vesicle, *GDF15* Growth differentiation factor-15

^*^P < 0.05

^**^P < 0.001

### Correlation of EV-GDF15 and serum GDF15 with clinical or biochemical variables

Spearman correlation analysis showed that EV-GDF15 levels were positively correlated with FPG and HbA1c (*R* = 0.619, *P* < 0.001; *R* = 0.574, *P* < 0.001, respectively); the same tendency was also observed between serum GDF15 and FPG and HbA1c (*R* = 0.349, *P* < 0.001; *R* = 0.352, *P* < 0.001, respectively). As expected, EV-GDF15 levels were positively correlated with serum GDF15 (*R* = 0.375, *P* < 0.001). In addition, EV-GDF15 levels were positively correlated with Hcy (*R* = 0.191, *P* = 0.039) and negatively correlated with TC (*R* = − 0.227, *P* = 0.014) and LDL-C (*R* = − 0.188, *P* = 0.043). Serum GDF15 levels were positively correlated with age (*R* = 0.502, *P* < 0.001), Hcy (*R* = 0.221, *P* = 0.017), SBP, and DBP (*R* = 0.358, *P* < 0.001; *R* = 0.277, *P* = 0.003, respectively) (Additional file 1: Table S1). More importantly, the results of multiple stepwise regression analysis showed that FPG and HbA1c were independently positively associated with EV-GDF15 levels (normalized *β* = 0.357, *P* < 0.001; normalized *β* = 0.409, *P* < 0.001, respectively), even after adjusting for gender, age, BMI, SBP, TG, HDL-C, LDL-C, hs-CRP, smoking, and alcohol consumption (Table 2).

**Table 2:** Multivariate stepwise regression analysis of the association between FPG and HbA1c and EV-GDF15 levels

Variable	Normalized β	*t*	*P*
FPG (mmol/L)	0.357	3.855	< 0.001**
HbA1c (%)	0.409	4.625	< 0.001**

Independent variables originally included gender, age, BMI, smoke, drink, SBP, TG, HDL-c, LDL-c, hs-CRP

*FPG* fasting plasma glucose, *HbA1c* glycated hemoglobin, *BMI* body mass index, *SBP* systolic blood pressure, *TG* triglyceride, *HDL-C* high-density lipoprotein cholesterol, *LDL-C* low-density lipoprotein cholesterol, *hs-CRP* high-sensitivity C-reactive protein, *EV* extracellular vesicle, *GDF15* Growth differentiation factor-15

### Relationship between the levels of EV-GDF15 and T2DM

We calculated the mean levels of EV-GDF15 and serum GDF15 in the enrolled population separately. The results showed that both levels were significantly higher in T2DM patients than in non-T2DM patients (EV-GDF15 levels, 13.68 (6.61–23.44) pg/mL vs. 5.56 (3.44–12.09) pg/mL, *P* < 0.001; and serum GDF15 levels, 1025.49 (677.87–1626.36) pg/mL vs. 675.46 (469.53–919.98) pg/mL, *P* < 0.001) (Additional file 1: Fig. S4). To present the relationship between the levels of EV-GDF15 and T2DM, furthermore, we analyzed the tendency of EV-GDF15 and T2DM. As shown in Additional file 1: Fig. S5, the proportion of T2DM patients (orange) increased from approximately 47.5 to 78.9% as EV-GDF15 levels increased from less than 6.01 pg/mL to the highest levels (≥ 17.25 pg/mL).

**Fig. 2:**
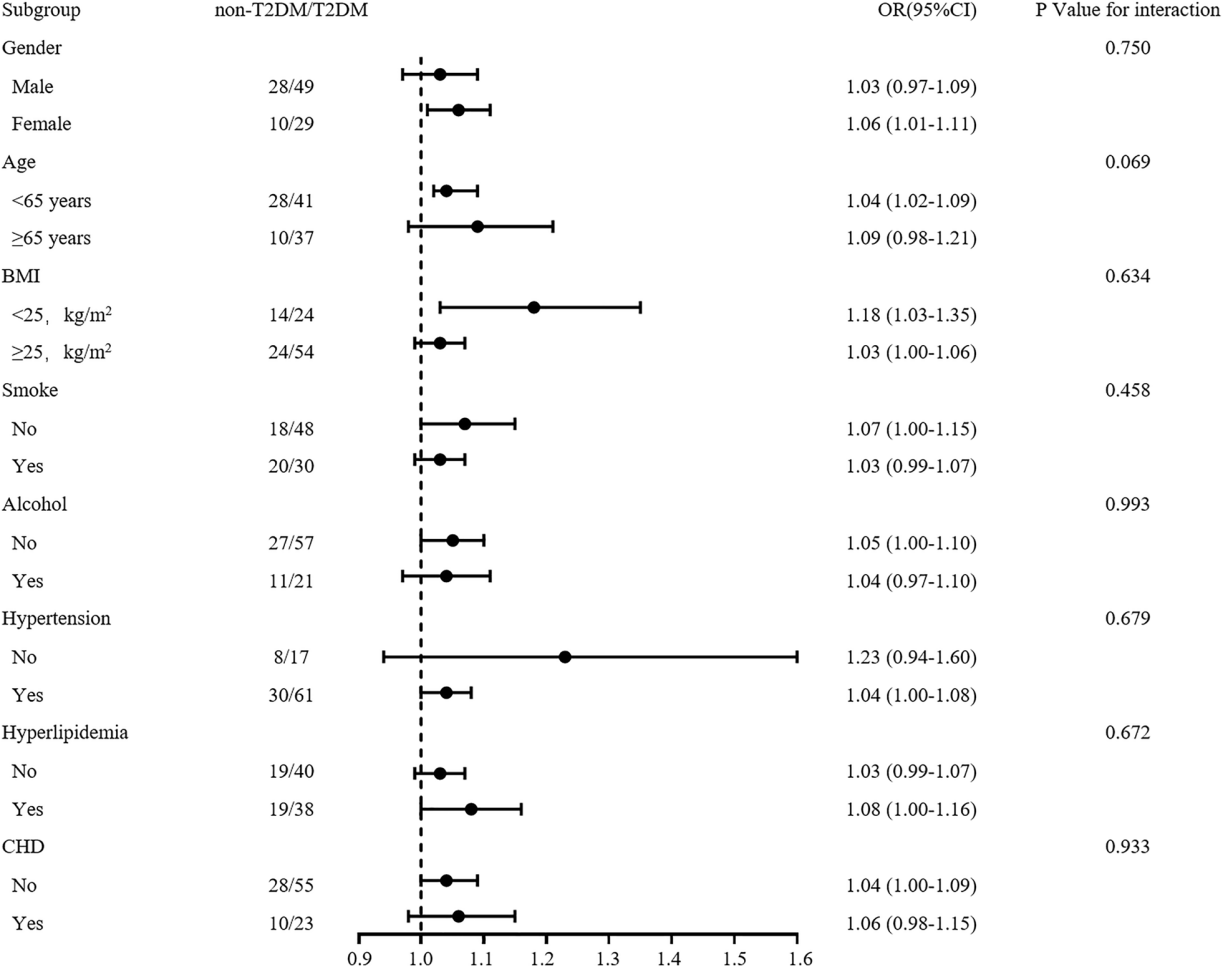
Subgroup analysis. Subgroup analysis for the association between EV-GDF15 levels and T2DM. *T2DM* type 2 diabetes mellitus, *BMI* body mass index, *CHD* coronary heart disease

Then we performed logistic regression analysis to further determine the relationship between EV-GDF15 levels and T2DM. It was observed that increased EV-GDF15 levels were significantly associated with a gradual increased risk of T2DM incidence when unadjusted or adjusted together with the indicated cofounder factors. More specifically, compared with the lowest tertile of EV-GDF15, the risk of T2DM increased by 206% in the middle tertile (OR = 3.06; 95% CI 1.02–9.19), and increased by 275% in the upper tertile (OR = 3.75; 95% CI 1.14 to 12.26) (Table 3). To further investigate the possibility of relevant baselines attributed to the association between serum EV-GDF15 levels and T2DM, we computed the odds ratios in the relevant subgroups and performed tests of interaction. No significant difference was observed for the association of serum EV-GDF15 with T2DM in the different groups of gender, BMI, age, smoking, alcohol consumption, hypertension, hyperlipidemia, and coronary heart disease (Fig. 2).

**Table 3:** Association between EV-GDF15 levels and T2DM

EV-GDF15	< 6.01(pg/mL) (*n* = 40)	< 17.25 (pg/mL) (*n* = 38)		≥ 17.25 (pg/mL) (*n* = 38)	
	ORs (95% CI)	ORs (95% CI)	*P*	ORs (95% CI)	*P*
Unadjusted	1.00 (Reference)	3.56 (1.35–9.41)	0.010*	4.15 (1.53–11.23)	0.005*
Model 1	1.00 (Reference)	2.93 (1.06–8.14)	0.039*	4.52 (1.58–12.92)	0.005*
Model 2	1.00 (Reference)	3.06 (1.02–9.19)	0.047*	3.75 (1.14–12.26)	0.029*

Model 1: adjusted for age, gender, and BMI

Model 2: adjusted for Model 1 + smoke, drink, DBP, HDL-c, LDL-c, TG, Hcy, hs-CRP

*T2DM* type 2 diabetes mellitus, *BMI* body mass index, *DBP* diastolic blood pressure, *HDL-C* high-density lipoprotein cholesterol, *LDL-C* low-density lipoprotein cholesterol, *TG* triglyceride, *Hcy* homocysteine, *hs-CRP* high-sensitivity C-reactive protein, *EV* extracellular vesicle, *GDF15* growth differentiation factor 15

It has been reported that metformin increased serum GDF15 levels [26]. Consistently, in this study, we also found that serum GDF15 levels were significantly increased in T2DM patients taking metformin compared with T2DM patients not taking metformin [851.31 (611.15–1237.18] vs. [1370.67 (723.22–1895.21), *P* = 0.034], but EV-GDF15 levels were not significantly different between the two groups [15.11 (9.60–21.07) vs. 10.77 (5.40–27.75), *P* = 0.293] Additional file 1: Fig. S6).

### The association of the combined levels of EV-GDF15 and serum GDF15 with T2DM incidence

**Fig. 3:**
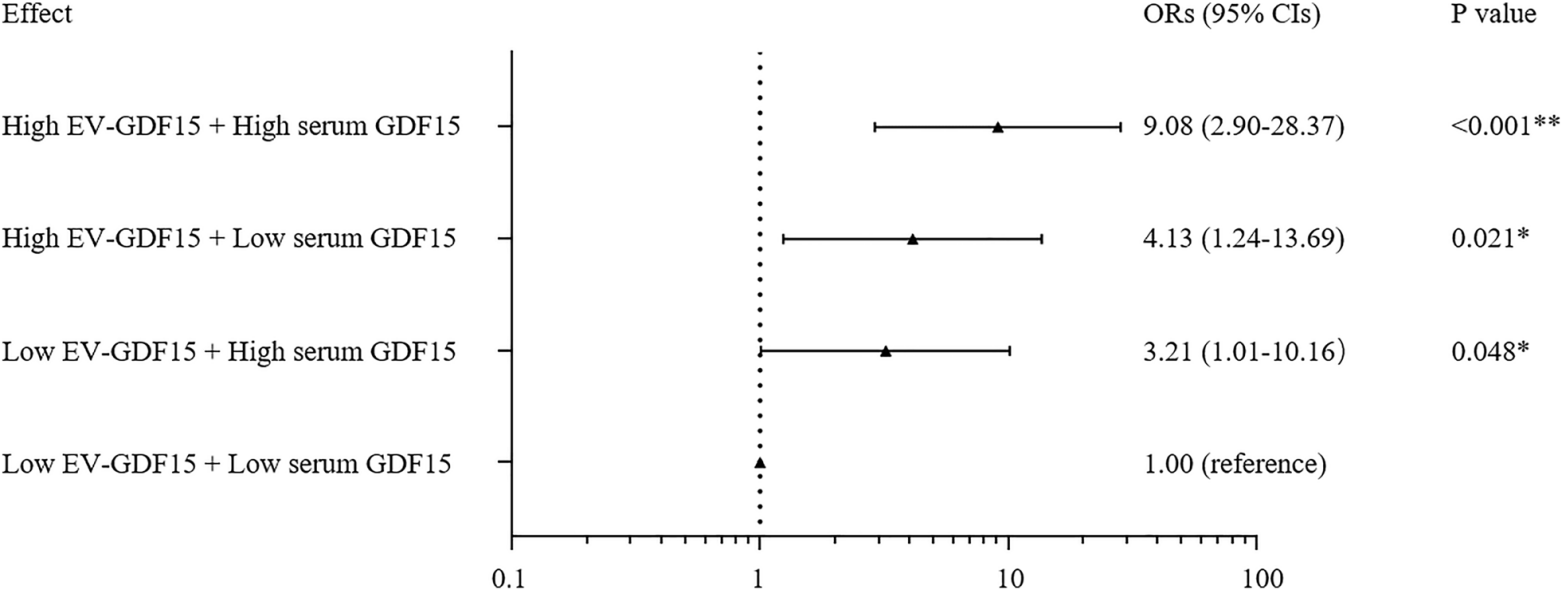
The combined association of EV-GDF15 levels and serum GDF15 levels with T2DM. The combined association of EV-GDF15 levels and serum GDF15 levels with T2DM after adjustment for gender, age, smoke, alcohol consumption, SBP, hs-CRP, TG, HDL-C, LDL-C. *BMI* body mass index, *SBP* systolic blood pressure, *HDL-C* high-density lipoprotein cholesterol, *LDL-C* low-density lipoprotein cholesterol, *hs-CRP* high-sensitivity C-reactive protein, *GDF15* growth differentiation factor 15

Taking low levels of EV-GDF15 (defined as < 10.48 pg/mL, below the median) plus low levels of serum GDF15 (defined as < 878.50 pg/mL, below the median) as reference, high levels of serum GDF15 (defined as ≥ 878.50 pg/mL, at or above the median) plus low levels of EV-GDF15 increased the risk of T2DM by 3.21-fold (95% CI 1.01–10.16). The combination of high levels of EV-GDF15 (defined as ≥ 10.48 pg/mL, at or above the median) and low levels of serum GDF15 increased the risk of T2DM by 4.13-fold (95% CI 1.24–13.69). Combining high levels of GDF15 of both sources increased the risk of T2DM by 9.08-fold (95% CI 2.90–28.37) (Fig. 3). The above results indicated that high levels of EV-GDF15 were more significantly associated with the presence of T2DM than high levels of serum GDF15, and that the combination of the two high levels of GDF15 from different origins has the highest correlation with T2DM incidence.

## Discussion

In the cross-sectional study, we presented that serum EV-GDF15 levels and serum GDF15 levels were significantly elevated in T2DM patients, and both of them were independently associated with FPG and HbA1c levels as well as T2DM after adjusting potential confounding factors. Furthermore, the combination of high levels of EV-GDF15 and serum GDF15 were significantly associated with the presence of T2DM more than either alone.

The representative indexes for identifying diabetes are FPG and HbA1c, but these glucose-dependent biochemical parameters are easily affected by a variety of factors. For instance, FPG levels can be influenced by hormones, drugs, alcohol intake, diurnal changes, and concomitant disease status [27], and HbA1c levels were significantly affected by anemia, hemoglobinopathy, liver diseases, alcohol, and vitamin C and vitamin E intake [28]. Therefore, the development of stable biomarkers to detect diabetes has great clinical significance. It has been reported that the levels of GDF15 were stable at room temperature for 48 h and even remained consistent over 4 freezing–thawing cycles [29]. These advantages, combined with the positive correlation of serum GDF15 with FPG, HbA1c, and insulin resistance, make circulating GDF15 a promising diagnostic biomarker for diabetes or diabetes-related diseases [23, 30–32].

EV cargoes change with disease progression and have successfully been used as biomarkers for numerous diseases. However, as for diabetes, most of the studies focused on the relevance of the overall EV quantitative levels rather than specific EV proteins [33]. There are a few clinical studies involving the correlation between EV-GDF15 and aging, advanced prostate cancer, and colon cancer [34–36], but clinical correlation studies between EV-GDF15 and T2DM have not been reported. In this study, we not only confirmed that serum EV concentrations were higher in patients with T2DM, but also accurately measured and highlighted the higher levels of GDF15 in serum EV in T2DM patients compared to non-T2DM patients.

Previous studies found that the levels of serum GDF15 were usually proportional to age [37] and SBP [38, 39], positively correlated with FPG, HbA1c, and Hcy in diabetes [40], while negative correlated with TC [41, 42], and BMI in monozygotic twin pairs [43]. Consistently, our results confirmed that serum GDF15 was positively correlated with age, FPG, HbA1c, SBP, and Hcy. However, there was no significant negative correlation between serum GDF15 and BMI as well as TC, even though the R-value was negative. As for EV-GDF15, there was a significant positive association with FPG, HbA1c, and Hcy, but a negative association with TC and LDL-C. Otherwise EV-GDF15 levels were not found to be in relation to age, BMI, or blood pressure, although they are all potential risk factors for diabetes. The reasons for the different correlation trends between EV-GDF15 and serum GDF15 and other biochemical indicators may be due to the different sources, as well as the limited number of participants. Additionally, the use of metformin may affect the judgment of the relationship between serum GDF15, EV-GDF15, and TG, LDL-C in diabetic patients since metformin enhanced serum GDF15 levels [26], but reduced TG and LDL-C levels [44], while did not alter the levels of EV-GDF15 according to our results.

## Limitations

This study for the first time elucidates the associations of EV-GDF15 and T2DM; however, there are several limitations in our study. This study cannot clearly describe the cause-and-effect relationship between EV-GDF15 and T2DM because of the cross-sectional nature, and more evidence from a prospective cohort study is required to demonstrate the causality. Meanwhile, we adjusted blood pressure and lipid levels in multivariate logistic regression analysis to compensate as much as possible for the defect of the limited sample size. Moreover, although we demonstrated an independent association between serum EV-GDF15 and diabetes, we still uncovered whether and how EV-GDF15 affects diabetes-related complications, and the exact mechanism by which EV-GDF15 is elevated in diabetes. The signaling pathways involved need deeply investigated. In addition, multi-center clinical study that need to be explored in the future could yield more representative enrollment ratio than the current single-center study. Finally, the trends in some diabetes risk indicators (e.g., BMI and age) and their correlation with EV-GDF15 did not seem reasonable due to the limited number of participants recruited for this study, but this did not negate the possible correlation of these parameters with EV-GDF15 in a large sample size.

## Conclusions

In conclusion, this is the first epidemiologic study to report a significant association between serum EV-GDF15 levels and T2DM. Future studies need to highlight the potential mechanisms of serum EV-GDF15 and its predictive value for outcome in T2DM patients.

## Supplementary Information

**Additional file1: Table S1.** Correlations of clinical variables with EV-GDF15 and serum GDF15. **Fig. S1.** Flow chart of patient selection.** Fig. S2. **Western blotting results showed the expression of CD63, CD81, and TSG101 in different protein fractions. **Fig. S3.** The tendency of FPG and HbA1c according to the tertiles of EV-GDF15. **Fig. S4.** Levels of EV-GDF15 and serum GDF15 in the enrolled population. **Fig. S5.** Prevalence of T2DM according to the tertiles of EV-GDF15. **Fig. S6.** The levels of serum GDF15 and EV-GDF15 in T2DM patients with or without metformin treatment. Serum GDF15 levels were significantly increased in metformin-treated T2DM patients compared to T2DM patients not taking metformin, but EV-GDF15 levels were not altered between the two groups.

## Data Availability

All original contributions presented in this study have been included in this article/supplementary material. The datasets used and analyzed in this study are not publicly available due to ethical reasons, but are also available from the corresponding author upon reasonable request.

## Abbreviations

T2DM: Type 2 diabetes mellitus
EV: Extracellular vesicle
GDF15: Growth differentiation factor-15
BMI: Body mass index
SBP: Systolic blood pressure
DBP: Diastolic blood pressure
FPG: Fasting plasma glucose
HbA1c: Glycated hemoglobin
TG: Triglycerides
TC: Total cholesterol
LDL-C: Low-density lipoprotein cholesterol
HDL-C: High-density lipoprotein cholesterol
Hcy: Homocysteine
hs-CRP: High-sensitive C-reactive protein
NTA: Nanoparticle tracking analysis
TEM: Transmission electron microscope
PBS: Phosphate buffered saline
CI: Confidence interval
