# Supplementary material for: Significant increase of serum extracellular vesicle-packaged growth differentiation factor 15 in type 2 diabetes mellitus: a cross-sectional study
Source: Eur J Med Res. 2023 Jan 19;28:37. doi: 10.1186/s40001-023-01009-6 (PMC9850700; doi:10.1186/s40001-023-01009-6)
Supplement: Supplementary file 1 — Additional file1: Table S1. Correlations of clinical variables with EV-GDF15 and serum GDF15. Fig. S1. Flow chart of patient selection. Fig. S2. Western blotting results showed the expression of CD63, CD81, and TSG101 in different protein fractions. Fig. S3. The tendency of FPG and HbA1c according to the tertiles of EV-GDF15. Fig. S4. Levels of EV-GDF15 and serum GDF15 in the enrolled population. Fig. S5. Prevalence of T2DM according to the tertiles of EV-GDF15. Fig. S6. The levels of serum GDF15 and EV-GDF15 in T2DM patients with or without metformin treatment. Serum GDF15 levels were significantly increased in metformin-treated T2DM patients compared to T2DM patients not taking metformin, but EV-GDF15 levels were not altered between the two groups. [file 40001_2023_1009_MOESM1_ESM.docx]

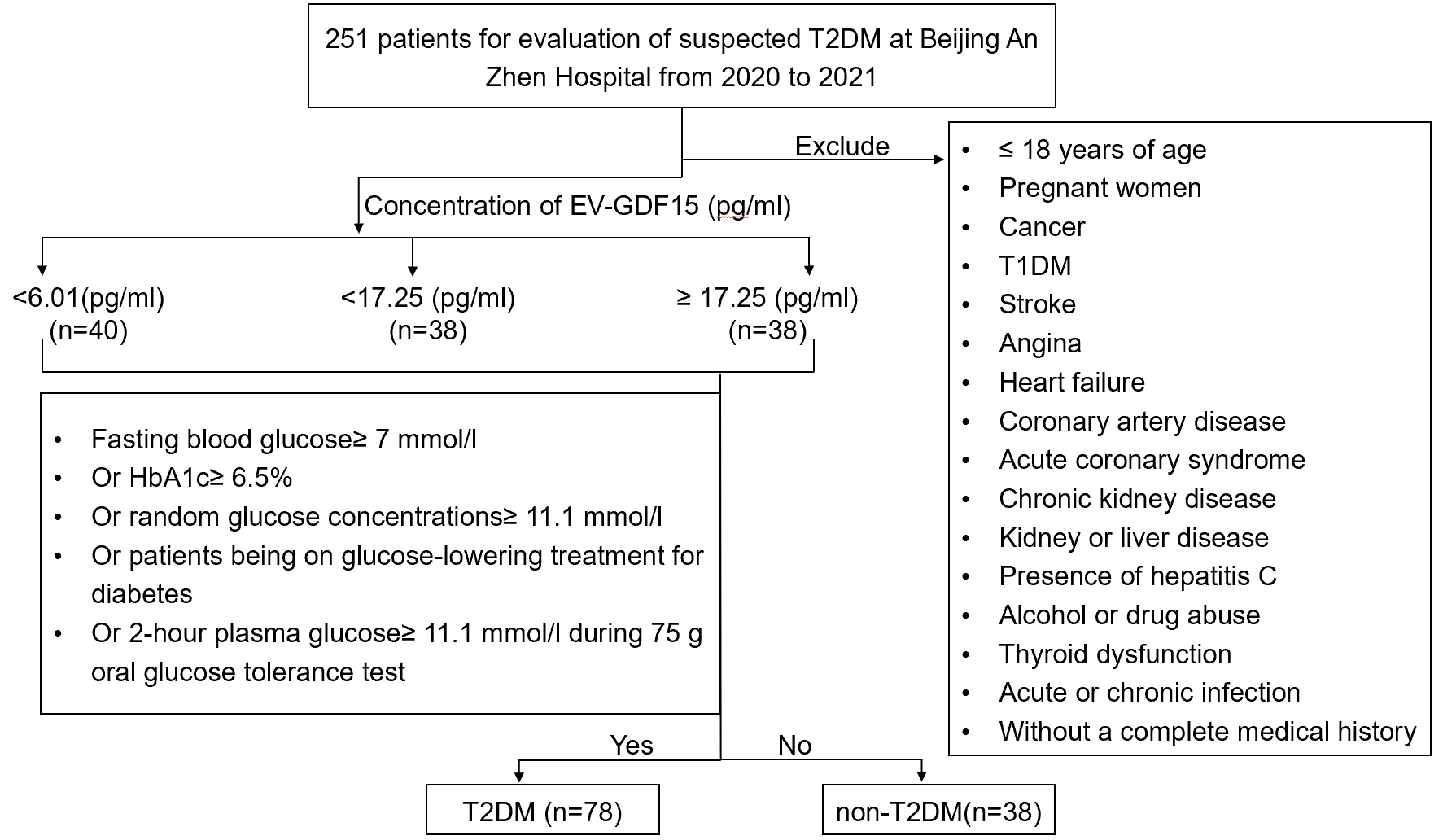


**Figure S1.** **Flow chart of patient selection.**

116 participants were consecutively enrolled including 38 non-T2DM controls and 78 T2DM patients. T2DM, type 2 diabetes mellitus; HbA1c, glycated hemoglobin.

**Figure S2. Western blotting results (uncropped images) showed the expression of CD63, CD81, and TSG101 in different protein fractions.** EVs, extracellular vesicles.


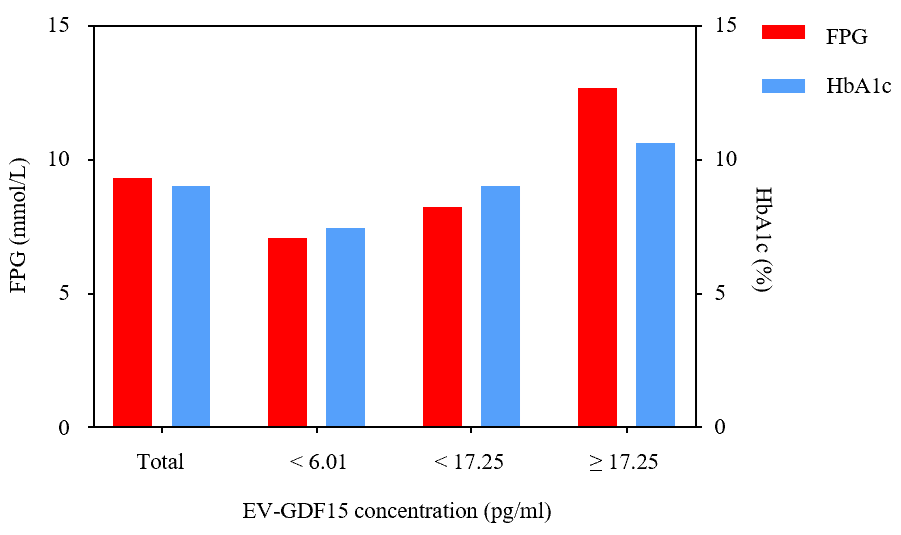


**Figure S3.** **The tendency of FPG and HbA1c according to the tertiles of EV-GDF15.** EV, extracellular vesicle; GDF15, Growth differentiation factor-15; FPG, fasting plasma glucose; HbA1c, glycated hemoglobin.


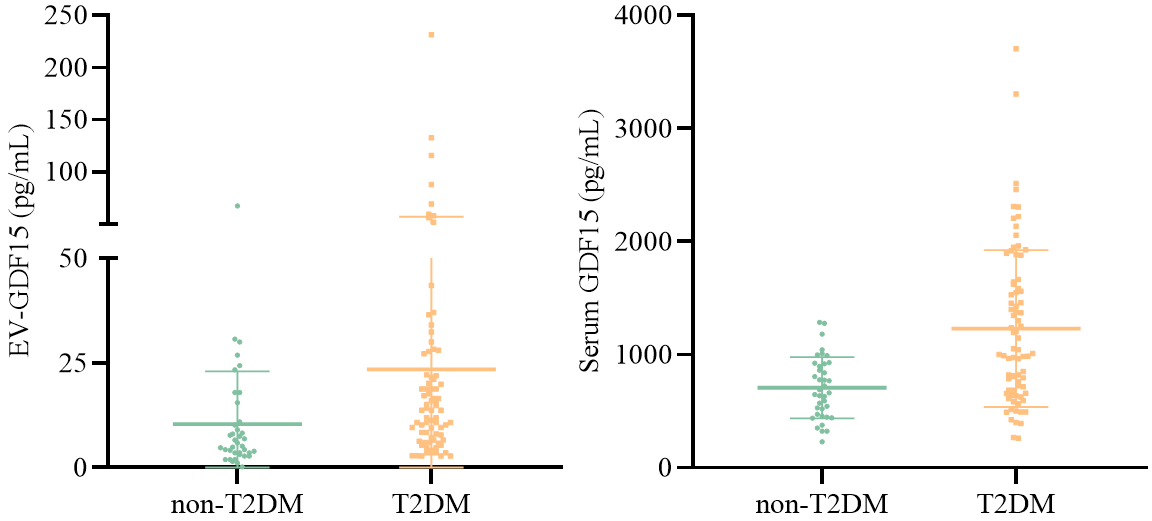


**Figure S4.** **Levels of EV-GDF15 and serum GDF15 in the enrolled population.** EV, extracellular vesicle; GDF15, Growth differentiation factor-15; T2DM, type 2 diabetes mellitus.


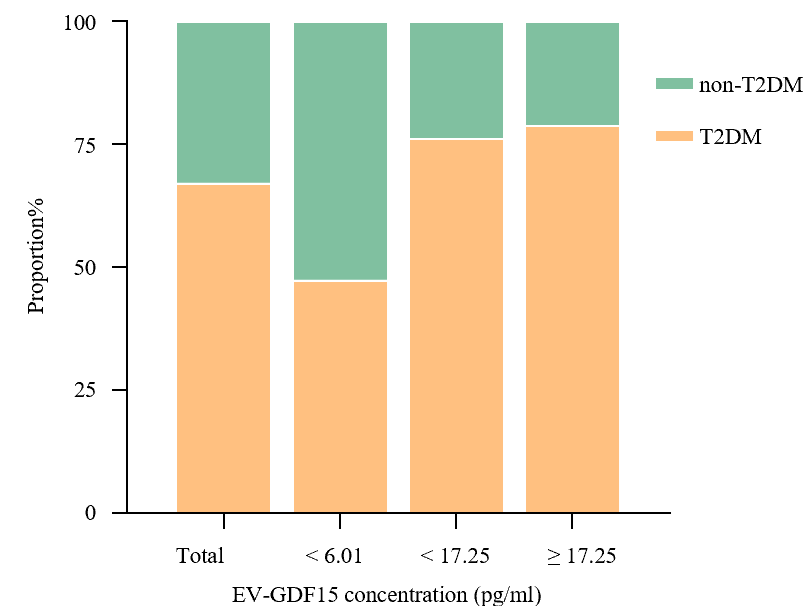


**Figure S5.** **Prevalence of T2DM according to the tertiles of EV-GDF15.**

EV, extracellular vesicle; GDF15, Growth differentiation factor-15; T2DM, type 2 diabetes mellitus.


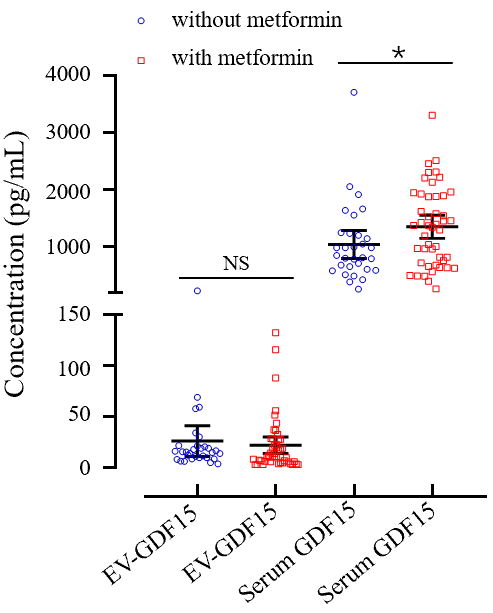


**Figure S6.** **The levels of serum GDF15 and EV-GDF15 in T2DM patients with or without metformin treatment.** Serum GDF15 levels were significantly increased in metformin-treated T2DM patients compared to T2DM patients not taking metformin, but EV-GDF15 levels were not altered between the two groups. ^*^*P* < 0.05 vs. without metformin. Abbreviation: EV, extracellular vesicle; GDF15, Growth differentiation factor-15; T2DM, type 2 diabetes mellitus.

**Table S1.** Correlations of clinical variables with EV-GDF15 and serum GDF15.

|  | **EV-GDF15** | |  | | **Serum GDF15** | | |  |
| --- | --- | --- | --- | --- | --- | --- | --- | --- |
|  | **Correlation coefficient** | **P value** | |  | | **Correlation coefficient** | **P value** | |
| **Age (year)** | 0.002 | 0.981 | |  | | 0.502 | <0.001** | |
| **BMI (kg/m^2^)** | 0.042 | 0.652 | |  | | -0.164 | 0.080 | |
| **SBP (mmHg)** | 0.141 | 0.131 | |  | | 0.358 | <0.001** | |
| **DBP (mmHg)** | 0.080 | 0.392 | |  | | 0.277 | 0.003* | |
| **FPG (mmol/L)** | 0.619 | <0.001** | |  | | 0.349 | <0.001** | |
| **HbA1c (%)** | 0.574 | <0001** | |  | | 0.352 | <0.001** | |
| **TG (mmol/L)** | -0.177 | 0.057 | |  | | -0.067 | 0.473 | |
| **TC (mmol/L)** | -0.227 | 0.014* | |  | | -0.159 | 0.089 | |
| **LDL-C (mmol/L)** | -0.188 | 0.043* | |  | | -0.157 | 0.092 | |
| **HDL-C (mmol/L)** | -0.131 | 0.161 | |  | | -0.051 | 0.587 | |
| **hs-CRP (mg/L)** | 0.172 | 0.065 | |  | | 0.157 | 0.092 | |
| **Hcy (μmol/L)** | 0.191 | 0.039* | |  | | 0.221 | 0.017* | |
| **Serum GDF15 (pg/ml)** | 0.375 | <0.001** | |  | | / | / | |

Abbreviation: BMI, body mass index; SBP, systolic blood pressure; DBP, diastolic blood pressure; FPG, fasting plasma glucose; HbA1c, glycated hemoglobin; TG, triglycerides; TC, total cholesterol; LDL-C, low density lipoprotein cholesterol; HDL-C, high-density lipoprotein cholesterol; Hcy, homocysteine; hs-CRP, high sensitive C-reactive protein; EV, extracellular vesicle; GDF15, Growth differentiation factor-15. ^*^*P* < 0.05，^**^*P* < 0.001.
